# The Role of Imaging in Diagnosing Axial Spondyloarthritis

**DOI:** 10.3389/fmed.2018.00106

**Published:** 2018-04-17

**Authors:** Nikita Khmelinskii, Andrea Regel, Xenofon Baraliakos

**Affiliations:** ^1^Rheumathology Department, Hospital de Santa Maria, Centro Hospitalar Lisboa Norte, Lisbon Academic Medical Centre, Lisbon, Portugal; ^2^Rheumazentrum Ruhrgebiet Herne, Ruhr-University Bochum, Herne, Germany

**Keywords:** ankylosing spondylitis, axial spondyloarthritis, diagnosis, imaging techniques, inflammatory lesions, structural lesions

## Abstract

Imaging has a central role in the diagnosis, management, and follow-up of patients with axial spondyloarthritis (axSpA). For the early diagnosis of axSpA, magnetic resonance imaging is of utmost relevance. While no novel imaging techniques were developed during the past decade, improvements to the existing modalities have been introduced. This report provides an overview of the applications and limitations of the existing imaging modalities.

## Introduction

Axial spondyloarthritis (axSpA) is a chronic rheumatic inflammatory disease affecting primarily the spine and the sacroiliac joints (SIJs). The diagnosis “axSpA” comprises both stages of disease: from the “early” non-radiographic axSpA (nr-axSpA) to the “established” radiographic axSpA, known as ankylosing spondylitis (AS) (1). A strong correlation exists concerning the occurrence of the disease and the human leukocyte antigen B27 (2). The most prominent symptom in patients with axSpA is chronic inflammatory back pain (IBP). About 70–80% of patients with axSpA suffer from IBP while IBP is relatively uncommon in patients with back pain of other source (2, 3). Consequently, this led for the incorporation of IBP in the 1984 modified New York criteria for the diagnosis of AS, as one of the three clinical criteria (4). Furthermore, these criteria required the presence of advanced sacroiliitis on plain radiographs as a classic and necessary diagnostic hallmark of AS. Although the modified New York criteria are quite specific and perform well in patients with established disease, they showed to be too insensitive for diagnosing early stages of disease. An average delay of up to 7 years is reported since the onset of the first clinical symptoms and the final diagnosis of AS. The rather late development of definitive radiographic sacroiliitis and other changes on conventional radiography (CR) is the main factor for this diagnostic delay (5, 6). In the setting of early axSpA, imaging and in particular magnetic resonance imaging (MRI) are expected to enhance diagnostic accuracy. This notion is reflected by the 2009 Assessment of SpondyloArthritis Society (ASAS) classification criteria. For the classification of axSpA, these criteria comprise an imaging and a clinical arm. For the first time, the MRI of the SIJs is incorporated in the imaging arm as a major criterion. The sensitivity and specificity were reported as 66.2 and 97.3% by the authors (7, 8). This concept of axSpA and the ASAS classification criteria have promoted a better understanding of its broader disease spectrum (7, 8). Considerable symptom overlap between inflammatory and mechanical back pain and limited accessibility to clinical examination of the SIJs validate the importance of the imaging of the SIJs in the early recognition of axSpA, as most patients show involvement of the SIJs. Spinal changes usually represent more advanced stages of the disease. Only a minority of patients with AS is reported to have spinal changes without SIJ changes (9). Therefore, the involvement of the spine has not been part of any classification criteria for axSpA. Characteristic findings of axSpA include inflammatory, osteodestructive, or osteoproliferative changes in the SIJs and spine. Sacroiliitis, spondylitis, aseptic spondylodiscitis, and inflammation of the posterior elements of the spine are the typical inflammatory manifestations in the axial skeleton in axSpA (10), which later lead to new bone formation, such as syndesmophytes and ankylosis. Over the last decades, substantial progress within the field of imaging in axSpA provided with different techniques for the diagnosis, classification, assessment of disease activity, structural damage, and prognosis of patients with axSpA. Still, the gold standard for the assessment of structural damage in patients with axSpA is CR. Active inflammatory changes, not detected by CR or computed tomography (CT), are best detected by MRI. Several other imaging techniques are available but not systematically recommended for the diagnosis of axSpA. Overall, they should be used in a complementary fashion and according to individual indications (Table 1).

**Table 1:** Overview of the imaging techniques available for use in axial spondyloarthritis.

Techniques		Inflammatory/acute changes	Structural/chronic changes
Conventional radiography		−	+
Computed tomography		−	++
Spectral CT		+	+
Ultrasound		+	(+)
Scintigraphy		+	−
MRI	T1w	(+)	+
	STIR/T2FS/T1Gd	++	+
SPECT-CT		+	+
PET–CT, PET–MRI		+	+

*–, no diagnostic value; +, with diagnostic value; (+), limited diagnostic value; ++, with diagnostic value, gold standard. CT, computed tomography; MRI, magnetic resonance imaging; PET, positron emission tomography; SPECT, single-photon emission computed tomography; STIR, short tau inversion recovery sequence; T1Gd, gadolinium-enhanced T1-weighted sequence; T1w, T1-weighted sequence; T2FS, fat-saturated T2-weighted sequence*.

## Conventional Radiography

**Figure 1:**
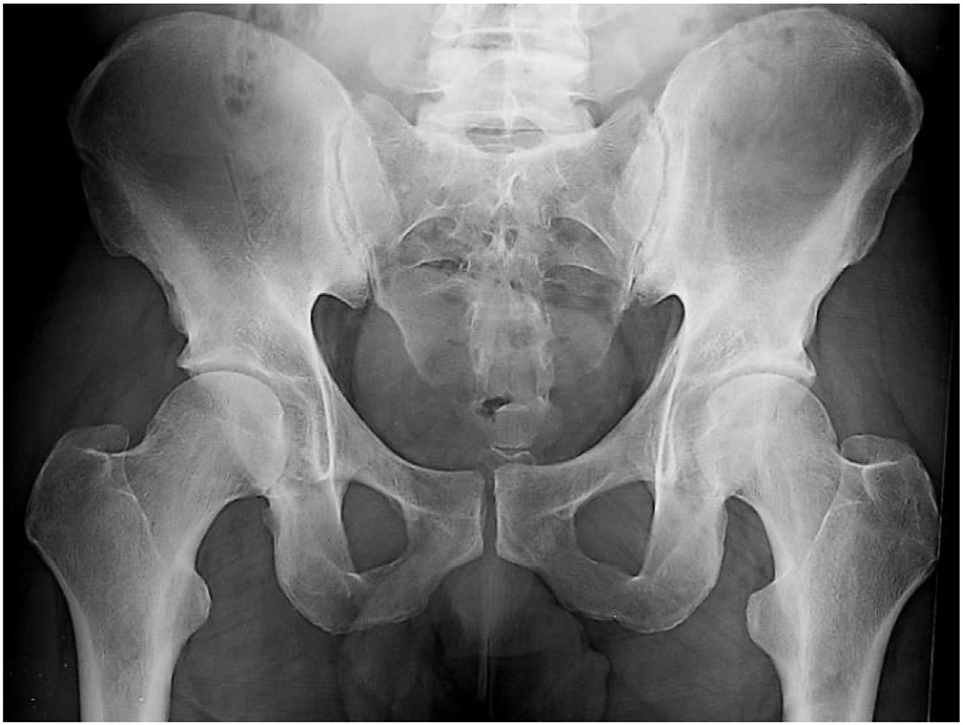
A 40-year-old male with a 3-year history of low back pain with short duration morning stiffness. Treatment with nonsteroidal anti-inflammatory drugs showed partial improvement. B27 is negative and C-reactive protein is 20 mg/L. Pelvic radiograph demonstrates suspicious changes of the right sacroiliac joint (SIJ) and minimal abnormalities of the left SIJ, not fulfilling the 1984 modified New York criteria for the diagnosis of ankylosing spondylitis.

Conventional radiography is an inexpensive, easy to generate, and well-established imaging technique with wide acceptance. In general, CR of the SIJs is the first recommended modality for the diagnosis of axSpA and remains the gold standard for the assessment of structural changes in the spine and SIJs (11). Despite the increasing role of MRI in diagnosing sacroiliitis, CR and MRI have equal weights in the classification of sacroiliitis according to the 2009 ASAS classification criteria for axSpA (7, 8). Typical radiographic findings are the result of osteodestructive or osteoproliferative changes caused by chronic inflammation. In the SIJs they include erosions, pseudo-widening, sclerosis, bony bridging, and/or SIJ ankylosis, and in the spine vertebral corner erosions, enthesophytes, vertebral squaring, sclerosis and erosions of the vertebral endplate, disk calcifications, spondylophytes, syndesmophytes, bony bridging, and/or intervertebral ankylosis. Significant intraobserver and interobserver variations have been reported due to difficulties in the interpretation of CR of the SIJs (12, 13). Several projections have been suggested for the visualization of the SIJs. The techniques of CR of the SIJs have been previously described (14). Pelvic radiography remains the cornerstone of diagnostic evaluation of axSpA in the clinical settings. A frontal projection of the SIJs is preferred. An anterior–posterior view of the SIJs is usually performed with the patient in the supine position and the tube angulated 15–30° in the cephalic direction (15). A posterior–anterior view in the prone position with 25–30° caudal angulation may also be used (15). These views ease the comparison of the two SIJs by exposing both joints on a single film. To enhance the visualization with overprojecting bone structures on frontal projections, supplementary oblique views have been proposed. These are performed as a separate radiograph of each joint, either in the supine or the prone position with the side of the body elevated 20–25° (15). Nonetheless, oblique views add minimal diagnostic value comparted to the anterior–posterior view, as proved by Battistone et al. (16). Radiography has low sensitivity for SIJ disorders. Therefore, patients with low back pain should not be routinely screened for chronic SIJ changes (17). In younger patients with high suspicion of axSpA—with IBP or morning stiffness, this may not be the case (10). Erosions in the iliac side of the SIJs are the earliest radiographic changes visualized in axSpA (18), with evidence suggesting that radiographic progression from nr-axSpA to AS may develop rather rapidly in approximately 10% of patients over 2 years (19, 20). The semiquantitative method of quantification of radiographic changes of the SIJs (Table 2) has been used for the diagnosis of AS according to the 1984 modified New York criteria (4) and classification of axSpA according to the 2009 ASAS classification criteria (7, 8). Although not included in any classification criteria for AS or axSpA, CR of the spine may support the diagnosis of axSpA in patients with indefinite SIJ changes when syndesmophytes are present. New bone formation, syndesmophytes, and ankylosis of the vertebral column are almost pathognomonic for axSpA (21). Syndesmophytes, characterized by their vertical growth in the outer lamellae of the annulus fibrosus or in the prediscal space between the annulus fibrosus and the anterior longitudinal ligament (9, 22, 23), are the best predictors of radiographic progression (21). Most of the data regarding radiological progression of axSpA pertains to CR (24, 25). Thus, it is regarded as the gold standard for the assessment of chronic and structural spinal lesions (26), as well as for the assessment of radiographic change in AS (21). For the purpose of quantification of radiographic changes of the spine in axSpA, several scoring systems have been developed: the Stoke AS Spine Score (SASSS) (27), the modified SASSS (28), the Radiographic AS Spinal Score (29), the Bath AS Radiology Index (23), and the Psoriatic Arthritis Spondylitis Radiology Index (30). Nevertheless, the main limitation of these scores is the low sensitivity to change before 2 years of follow-up (31). Furthermore, in the absence of therapeutic agents with disease-modifying properties, spinal radiography has limited use in the assessment of axSpA. For the purpose of early diagnosis of axSpA, relying solely on CR is inadequate and may delay treatment (Figure 1).

**Table 2:** Grading of radiographic sacroiliitis (4).

Grade	Definition or radiographic changes
0	Normal
1	Suspicious changes
2	Minimal abnormalities: small localized areas with erosion and sclerosis, without alteration in the joint width
3	Unequivocal abnormality: moderate or advanced sacroiliitis with 1 or more signs of erosions, sclerosis, widening, joint space narrowing, or partial ankylosis
4	Severe changes: total ankylosis

Computed tomography is even better than CR for the detection of erosions and new bone formation, but as the latter, it is unable to perceive inflammation and has particularly low sensitivity for the detection of bony and soft tissue changes found in early sacroiliitis (15). In cases of younger patients or those with symptoms of shorter duration MRI of the SIJs may be regarded as the first imaging method (11). Instead, combined imaging may yield the highest returns for depicting SIJ involvement in patients with suspected early axSpA, with sequential assessment of inflammation by MRI considered for those patients without structural changes on CR (32).

## Magnetic Resonance Imaging

**Figure 2:**
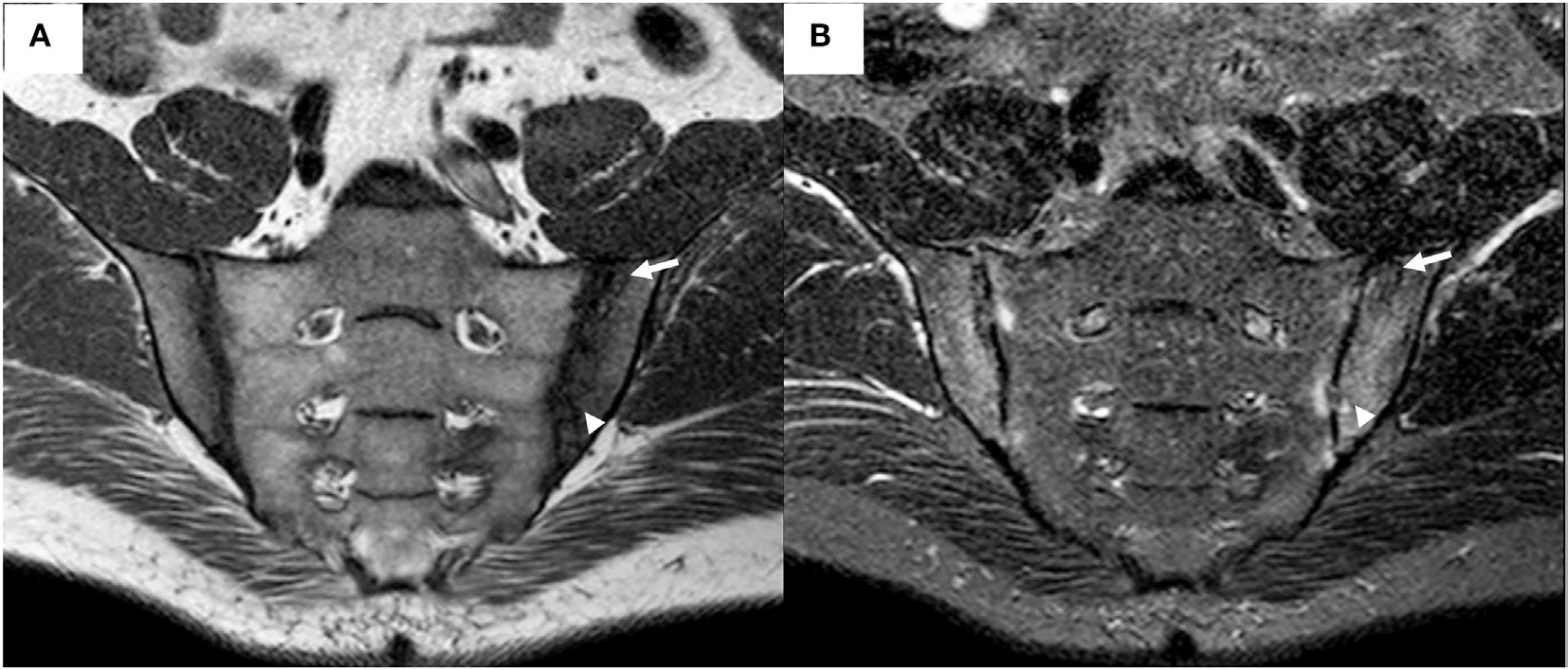
Magnetic resonance imaging (MRI) of the sacroiliac joints (same patient as depicted in Figure 1) demonstrates bilateral irregularity of the joint margins, and subchondral bone marrow edema appearing as a hypointense signal on T1-weighted (T1w) sequence **(A)** and as a hyperintense signal on short tau inversion recovery (STIR) sequence **(B)**, fulfilling the Assessment of SpondyloArthritis Society criteria for positive MRI for sacroiliitis. An area of subchondral sclerosis with hypointense signal on both sequences (arrow), and an active erosion with hypointense signal on T1w sequence and hyperintense on STIR sequence (arrowhead), are visible in the left ilium.

Daily management of axSpA was transfigured by MRI. Among the musculoskeletal imaging techniques, it is the only one able to detect both active inflammatory and structural lesions (Table 3), as well as their anatomical distribution. Therefore, MRI is particularly useful for the early diagnosis of axSpA (32), capable of detecting both bone marrow edema (BME) or osteitis and erosions before CR (33, 34). In addition, inflammation of the SIJs as detected by MRI correlates with histological and clinical finding in axSpA (35, 36). Thus, in the setting of suspected axSpA, when the diagnosis cannot be established based on clinical features and CR, assessment of the SIJs by MRI should be conducted (Figure 2) (11). In addition, the lack of radiation exposure during MRI is an obvious advantage, making it particularly useful in children, young female patients, those with repeated past radiation exposure, and for repeated imaging during follow-up. Some of its limitations include the higher cost than other imaging techniques, restricted availability, intolerance in patients with claustrophobia and active axSpA due to long procedure duration, contraindication in patients with pacemakers or metal implants, and false positives. In patients with already established axSpA, the role of MRI in clinical practice is restricted to the differential diagnosis of exacerbations of spinal symptomatology in patients with previously stable clinical disease. BME as a sign of osteitis in the axial skeleton detected by MRI is particularly useful for the diagnosis of axSpA (37). Yet, it is not a specific feature for axSpA occurring in other inflammatory and non-inflammatory conditions. In the SIJs, other active inflammatory lesions associated with axSpA as shown by MRI, though rare as isolated features, include synovitis, enthesitis, and capsulitis. BME is depicted with high-intensity signal by short tau inversion recovery (STIR) sequences, while osteitis, synovitis, and capsulitis are detected by gadolinium-enhanced T1-weighted (T1Gd) sequences, with or without fat suppression. Typical structural lesions of the SIJs in axSpA are subchondral sclerosis, erosions, backfill and subchondral fat metaplasia, and bony bridges/ankylosis. For the detection of structural changes, T1-weighted (T1w) sequences are usually sufficient. Fat metaplasia is of particular interest as it cannot be detected by CR (34). Indicating areas of previous inflammation, backfill (fat metaplasia in excavated erosions), a specific sign of axSpA (38), and subchondral fat metaplasia are associated with radiographic progression (39). In the spine, typical inflammatory findings by MRI include spondylitis, inflammation of the facet joints, and aseptic spondylodiscitis (40). As in the SIJs, fat metaplasia in the vertebral corners is associated with radiographic progression (41, 42). Similar to MRI of the SIJs, clinical findings in patients with recent onset IBP, i.e., the site of pain, are associated with inflammation on MRI at the same sites (43).

**Table 3:** Characteristic lesions in the sacroiliac joints and the spine of patients with axial spondyloarthritis as depicted by magnetic resonance imaging (37, 40).

Inflammatory changes	Structural changes
**SIJs**	
Sacroiliitis—bone marrow edema/osteitis in one or both part of the sacroiliac joint (iliac or sacral)	Subchondral sclerosis
Synovitis	Erosions
Capsulitis	Backfill/subchondral fat metaplasia
	Bony bridges
Enthesitis	Ankylosis

**Spine**	
Anterior/posterior spondylitis—bone marrow edema/osteitis mainly in the vertebral corners	Fat metaplasia
Spondylodiscitis	Erosions
Arthritis of costovertebral joints	Syndesmophytes
Facet joints arthritis	Ankylosis
Enthesitis of spinal ligaments	

### Definition of ASAS Positive MRI

According to the ASAS definition of positive MRI for sacroiliitis in axSpA (Table 4), the presence of BME/osteitis reflecting active inflammation, preferably located in the subchondral/periarticular region, is regarded as essential (37). The nonexistence of a clear quantitative requirement is the main limitation of this definition. Since several other conditions may present with similar inflammatory lesions to those seen in axSpA, the location of BME and its extension is of particular importance for this definition. The high prevalence of non-specific SIJ BME was recently confirmed (44), questioning the specificity of SIJ BME as the single MRI lesion able to distinguish patients with axSpA from those with non-SpA back pain. The development of MRI scoring systems may be necessary to increase confidence in the diagnosis (45). The ASAS criteria do not include other inflammatory lesions as well as structural lesions. Compared to the ASAS MRI criteria, diagnostic criteria that include structural lesions in the definition of positive MRI for sacroiliitis have lower sensitivity, lower specificity, or require further validation (46–48). In spite of the lack of data regarding their classification utility, structural lesions might assist in the recognition of patients with suspected axSpA and enhance the confidence in the classification of axSpA (45, 49). Specifically, erosions and fat metaplasia should be considered to be of importance for this purpose. In the spine, typical lesions of axSpA, but not pathognomonic as they can also occur in other diseases, include inflammatory and structural corner-based lesions. As such, for the ASAS definition of positive spine MRI, BME located in the corners of the vertebral bodies in three or more sites is considered highly suggestive of axSpA (40). Also, fat metaplasia in several vertebral corners is typical for axSpA. The likelihood of these findings being related to axSpA is increased in the younger age group, where the importance of degenerative changes in the differential diagnosis is decreased. Thus, MRI lesions at the spine demonstrate similar sensitivity and specificity to those at the SIJ (50). In recent work by Weber et al., none of the previously suggested criteria for positive spine MRI was found useful for the diagnosis of nr-axSpA (51). A threshold of six or more corner inflammatory lesions was proposed to increase the diagnostic utility of spine MRI in clinically suspected nr-axSpA. Showing no consistent beneficial effect for the classification of axSpA, MRI features of axSpA on spine are to date not included in the ASAS criteria.

**Table 4:** Features of an Assessment of SpondyloArthritis Society definition of positive magnetic resonance imaging for sacroiliitis (37).

Active inflammation of subchondral or periarticular bone marrow
Active inflammation is defined as bone marrow edema on short tau inversion recovery sequences or osteitis on gadolinium-enhanced T1-weighted sequences
Two or more lesions must be present on the same coronal slice or a single lesion must be visible on two consecutive slices
Other inflammatory features of axial spondyloarthritis, such as synovitis, enthesitis, and capsulitis are believed to be rare in the absence of bone marrow edema and in isolation are not sufficient for diagnosis

### MRI Protocol

Complementary sequences, a semicoronal T1w sequence and either a STIR or fat-saturated T2-weighted (T2FS) sequence, should be included in the routine evaluation of the SIJs by MRI. Because the semicoronal plane does not allow the visualization of the ventral and dorsal margin of the cartilaginous portion of the SIJs, and this limits the assessment of normal anatomy, variants, or abnormalities (35, 52), an additional semiaxial STIR or T2FS should be performed (53). Apart from allowing concurrent assessment of the cartilaginous and ligamentous compartments of the SIJs (54–56), the semiaxial might reduce misinterpretation of partial volume artifacts between both compartments as erosions (57). Yet, MRI assessment of the ligamentous portion of the SIJs adds no additional confidence in the diagnostic accuracy of nr-axSpA (58), and most of the proposed scoring modules to systematically assess SIJ MRI in axSpA are based on the cartilaginous joint compartment alone (33, 59–62). The T1Gd sequence is capable of detecting areas of increased vascularization due to increased diffusion of gadolinium into the interstitial space and synovitis thus being considered more sensitive than STIR (63). Still, it is time consuming, costly, and does not enhance the diagnostic utility of SIJ MRI over STIR or T2FS sequences (63, 64), therefore not being routinely recommended. The same sequences recommended for the evaluation of the SIJs are applied for the evaluation of the spine. A sagittal T1w sequence and either a STIR or T2FS are used, and should cover the entire vertebral body including the posterior-lateral elements (facet joints, costovertebral and costotransverse joints, spinous process), as they are frequently affected in AS (65). Although they may provide supplementary data, additional transverse and coronal sequences are not systematically recommended.

## Computed Tomography

**Figure 3:**
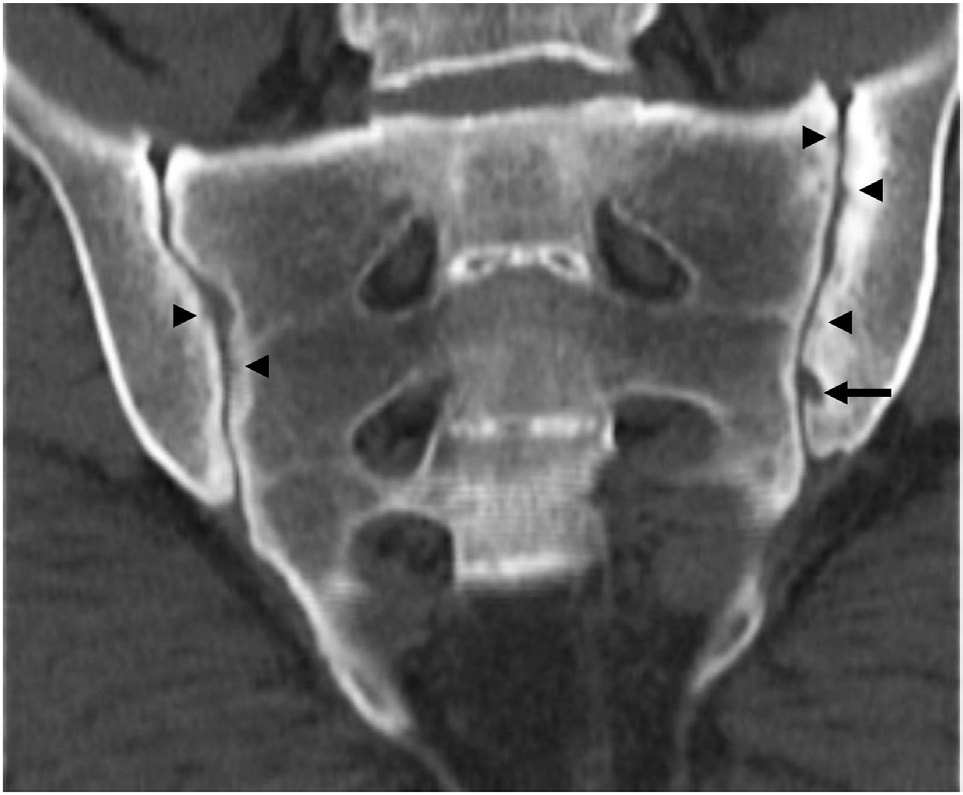
Computed tomography of the sacroiliac joints (same patient as depicted in Figures 1 and 2) demonstrating subchondral sclerosis predominantly of the left ilium, multiple bilateral small erosions (arrowheads), and a large erosion of the left ilium (arrow).

For the detection of SIJ structural lesions in axSpA, CT was shown to be superior then radiography (Figure 3) (66, 67). The ability of multi-planar assessment of anatomic structures by cutting them in slices is of particular importance in the area of the SIJs because of their irregular S-shaped orientation and the partly overlapping sacral and iliac joint structures. Therefore, CT has been widely used for imaging the SIJ for optimal analysis from the synovial joint space to the ligament compartment. It still has a place in the diagnosis of sacroiliitis, in patients with negative CR and when MRI cannot be performed, although the latter is diagnostically preferable and with no radiation risk. For the diagnosis of sacroiliitis, semicoronal CT is preferred. Comparing to axial CT semicoronal technique permits an overview of the cartilaginous and ligamentous portions of the SIJ with less radiation dose—6–8 contiguous 5-mm slices for the semicoronal CT and 14–16 contiguous 5-mm slices for the axial CT (68). Typical changes for sacroiliitis at CT encompass joint erosions, subchondral sclerosis on both sides of the joint, and ankylosis (69). Also, joint space narrowing and pseudo-widening are considered indicative of sacroiliitis while other features, such as indistinct articular margins, mild periarticular osteoporosis, and non-specific iliac sclerosis are of equivocal nature (70). However, similar to CR, CT findings may be misleading in elderly patients because subchondral sclerosis of the SIJs, particularly in the iliac part is due to aging, which is similar to joint space narrowing. As a solitary diagnostic sign only large or multiple erosions are reliable (70). An overall grading according to the modified New York criteria for CR has been used for the classification of sacroiliitis on CT. Still, these criteria should not be used with CT (70). A semiquantitative grading for CT abnormalities and a practical classification of sacroiliitis on CT have been proposed (54, 70). When compared to MRI, specifically for the detection of sclerosis, bone production, and chronic bone changes in the ligamentous portion of the joint, CT was reported to be superior (54). Yet, the radiation exposure of CT needs to be taken into consideration, even in repeated low-dose CT examinations. Therefore, for the assessment of patients with low back pain and suspected axSpA in daily practice, CT is not recommended.

### Spectral CT

Spectral CT, an emerging imaging modality, enables the quantification of the relative water and calcium contents in the bone *via* the acquisition of base material decomposition images (71). It is able to depict structural findings of chronic sacroiliitis and to detect active sacroiliitis in patients with axSpA, allowing qualitative and quantitative assessments of BME. In a study by Zhang et al. with 76 axSpA patients, spectral CT could demonstrate BME, bone sclerosis, and erosions in the absence of similar MRI findings (71). Early soft tissue inflammatory findings (i.e., capsulitis, enthesitis, and synovitis) could not be identified by this imaging method (71).

## Ultrasound

Recently, ultrasound (US), a noninvasive imaging modality, was shown to have high sensitivity for assessing soft tissue lesions in patients with inflammatory disorders. For the detection of enthesitis in patients with SpA, it showed to have higher sensitivity than MRI (72–74). In patients with axSpA and IBP, the site of pain may be located by US, thus supplementing the physical examination (75). Only the superficial part of the SIJs is accessible to visualization by US including the surrounding soft tissue structures and the posterior stabilizing ligaments, while the cartilaginous portion is inaccessible by this imaging modality. It has also been used to guide SIJ corticosteroid injections, particularly where these appear to be the primary affected joints (76–82). In a study involving 45 patients with axSpA, Spadaro et al. reported joint effusion using high-resolution US in 38.9% of SIJs of patients with axSpA and in 1.7% of SIJs of controls (75). In this study, SIJ effusion was detected in 78.6% of patients with axSpA with IBP and in 29.4% of patients with axSpA without IBP, therefore, revealing a significant association between the presence of IBP and SIJ effusion (75). Similarly, Duplex and color Doppler US have been found to detect SIJ and spine inflammation in AS patients, sensitive to change in response to treatment, based on increased vascularization and decrease resistive index (a measure of vascularity) in the posterior part of the SIJs and the spine (83, 84). In another study, which included 43 patients with axSpA with IBP and active sacroiliitis on MRI, unenhanced color Doppler US detected only 19% while microbubble contrast-enhanced color Doppler US 95% of these patients (85). Accordingly, the use of a contrast medium yielded higher sensitivity though with a somewhat lower specificity for the detection of active sacroiliitis. These finding suggest that US might be capable for assessing disease activity with limited value as a diagnostic tool. While US assessment is safe, noninvasive, comparably cheap and conveys no radiation, it is highly operator-dependent and influenced by the quality of the US equipment.

## Radionuclide Methods

### Bone Scintigraphy With Technetium-99 Labeled Methylene Diphosphate

By demonstrating increased radionuclide uptake in the areas of accelerated bone turnover, bone scintigraphy may be used as a screening method to detect bone metabolism due to any cause, including inflammation (86). Quantitative interpretation of the result is possible by comparing the intensity of radionuclide uptake in the area of interest with that of an adjacent background structure (86, 87). Although it has been used in the detection of acute sacroiliitis for the early diagnosis of axSpA, it is of limited value for this purpose. A review by Song et al. on the performance of bone scintigraphy showed an overall sensitivity of about 50% and specificity not higher than about 80% for the diagnosis of sacroiliitis (88). Higher specificity is reported for unilateral sacroiliitis and quantitative bone scintigraphy (calculation of the SIJ to sacrum uptake ratio), however, both with very low sensitivity (86, 87, 89). Furthermore, as with CT, the radiation exposure of bone scintigraphy limits its daily use in patients with suspected axSpA.

### Bone Scintigraphy With Technetium-99 Labeled Human Immunoglobulin and Anti-TNF-α

Few pilot studies reported the use of immunoscintigraphy with radiolabeled human immunoglobulin or anti-TNF-α in the detection of active sacroiliitis in patients with axSpA. Their results suggest that immunoscintigraphy may have value in the diagnosis of active sacroiliitis (90–92). Lacking confirmation in longitudinal studies, scintigraphy with radiolabeled anti-TNF-α may display good correlation with MRI findings and biological treatment response (92).

### Single-Photon Emission Computed Tomography (SPECT) and Combined SPECT-CT Imaging

The sensitivity of bone scintigraphy may be increased by using SPECT which allows slice-by-slice three-dimensional radionuclide uptake analysis, a particularly useful possibility in the study of the anatomically complex SIJs. Also, compared to bone scintigraphy, SPECT has been proven to be superior in quantifying the SIJ to sacrum ratio (93). The specificity of SPECT in clinical practice may further be increased by hybrid SPECT-CT modalities, which combines functional and anatomical imaging allowing anatomical localization and exclusion of physiological uptake for better characterization of equivocal lesions (94). In a recent study involving 20 patients with early axSpA, meeting the Amor criteria with minimal or no change in plain radiography, SPECT with low-dose CT of the SIJs was shown to be more useful in identifying early sacroiliitis compared with bone scintigraphy, with sensitivity of 80% and specificity of 84% for the diagnosis of sacroiliitis (95). Further research comparing SPECT-CT to MRI for the early diagnosis and follow-up of axSpA is required. Careful consideration is needed regarding the potential of follow-up imaging with SPECT-CT and the risk associated with repeated radiation exposure (95).

## More Modern Imaging Techniques

### Novel MRI Modalities

During the last few years, new MRI modalities have been used in the diagnosis and follow-up of axSpA patients. Advances in MR practice such as the use of multichannel systems and multiple coils now allow to perform whole-body MRI (wbMRI) (96). This method allows visualization of inflammation and assessment of all peripheral joints and axial joints (96, 97), thus allowing better characterization of the inherent variability of the disease manifestations in SpA. “True” wbMRI consists of coronal T1w and STIR sequences covering the entire spine, shoulder girdle, arms, anterior chest wall, pelvis including the SIJs and the lower extremities, while whole-spine MRI comprises sagittal sequences covering the entire spine with additional semicoronal sequences of the SIJs (97). Being sensitive in detecting and localizing inflammatory lesions in several sites, this modality is mostly helpful for the assessment of enthesitis (98, 99). As a “one-stop shop” modality for the evaluation of the spine, SIJs, joints and entheses, wbMRI may contribute for the early diagnosis of SpA and was shown to detect active inflammation and structural changes in active nr-axSpA and AS (96, 100). Sensitivity to change of wbMRI after treatment has been assessed in a few studies (101, 102). Of importance, wbMRI exhibits high readability for the spine, SIJs, and proximal peripheral joints, and when compared to conventional MRI, wbMRI exhibits moderate to strong agreement, depending on the image acquisition protocol (99, 103, 104). While promising as a screening tool for initial global assessment of the inflammatory and structural lesions and subsequent follow-up of patients with SpA, clinical implementation of wbMRI should be preceded by more research including optimization of image acquisition. Other MRI methods addressed the possibility of increasing the sensitivity of MRI to detect early osteitis and bone erosions. In a relatively new MRI modality, diffusion-weighted MRI (DWI), the image contrast is yielded by the random motion of the water molecules in different biological tissue environments, within the cellular and extracellular tissue compartments, providing both quantitative [apparent diffusion coefficient (ADC)] and qualitative functional information (105). Variations of the intra/extracellular ratio are responsible for the altered signal of inflammatory lesion. DWI was shown to identify active sacroiliitis based on conventional MRI on qualitative analysis, and to differentiate active from inactive sacroiliitis by quantitative ADC measurements (106). Gezmis et al. showed diffusion contrast alteration on DWI in patients with axSpA with no BME of the SIJs on STIR MRI (107). Similarly, Sahin et al. suggested that quantitative DWI may be complementary to conventional MRI for detection of osteitis, despite lower spatial resolution (108). Preliminary data with high-resolution MRI show an increased detection rate of erosions on the SIJs when compared to conventional MRI and low-dose CT (109).

### Positron Emission Tomography (PET) and Combined PET–CT, PET–MRI Imaging

By providing imaging of the functional tissue changes in the whole body, PET may be used for early detection of inflammation. The combination of PET and CT permits the integrated visualization of both early inflammatory and early structural lesions. Several PET tracers have been studied in axSpA: the glucose analog [^18^F]-fluoro-2-deoxy-d-glucose ([^18^F]FDG), the macrophage tracer PK11195 [(R)-1-(2-chlorophe-nyl)-*N*-methyl-*N*(1-methyl-propyl)-3-isoquinoline carboxamide] ([^11^C](R)PK11195), and the bone tracer of osteoblastic activity [^18^F]fluoride ([^18^F]F) (110). For the detection of sacroiliitis in patients with AS, [^18^F]FDG and [^11^C](R)PK11195 PET–CT was proved to be of limited value with inconsistent results in two studies (110). However, [^18^F]F PET-CT demonstrated sensitivity of 80% and specificity of 77% for the detection of sacroiliitis in active AS patients (111, 112). By demonstrating bone activity, [^18^F]F may reflect bone formation rather than inflammation and may be of little value for the diagnosis of nr-axSpA (113). Furthermore, lesions detected by [^18^F]F PET-CT have modest correlation with BME on MRI of the spine and SIJs in patients with AS (110, 114). The depiction of osteoblastic activity in the spine and SIJs in patients with AS by [^18^F]F was also assessed with PET–MRI. In a pilot study with active AS patients, Buchbender et al. showed that BME rather than structural lesions is correlated with osteoblastic activity (115). Although promising, the definite value of [^18^F]F PET-CT and PET–MRI for the diagnosis of axSpA and as a tool for assessment of AS activity requires confirmation, as well as the possible relation between inflammation on MRI, increased osteoblastic activity on [^18^F]F PET, and subsequent syndesmophyte formation.

## Differential Diagnoses

Although the existing definitions for positive MRI of the SIJs (37) and the spine (40) are still valid, it should be emphasized that BME of the SIJs or spinal structures is not pathognomonic to axSpA and may also occur in other inflammatory and non-inflammatory conditions. The most important differential diagnoses in the SIJs are infectious sacroiliitis, osteitis condensans ilii and extensive sclerosis, diffuse idiopathic skeletal hyperostosis (DISH), and pelvic fractures. In infectious sacroiliitis, inflammation often covers beyond anatomical borders and frequently extending to the surrounding soft tissue (116). MRI detects early signs of infection, while CR is usually normal in the first few weeks (10). Extensive sclerosis, at either side of the SIJ can lead to misdiagnosis of AS. Joint margins should be assessed for the occurrence of erosions and for joint width. With typical shape and location, osteitis condensans ilii is depicted as a triangular-shaped area of sclerosis of the iliac side of the SIJ, on MRI as on CR or CT (117). A rim of BME may be seen adjacent to the sclerotic area. It is frequently seen in middle-aged women after pregnancy, although it can rarely occur in men. In DISH, typical findings include irregularly shaped SIJs, sclerosis, ossification of the joint capsule, and bony bridges crossing both sides of the joint, mimicking sacroiliitis. Such changes, however, do not occur in young patients. Fractures and bone tumors, such as plasmacytoma or osteosarcoma, may cause reactive lesions with BME/osteitis-like appearance on MRI. Particularly, insufficiency fractures of the sacrum may present with low back pain and as the fracture line is not always visible, may lead to a misdiagnosis. In these instances, CT may provide valuable information. Small areas of BME along the SIJ may be found in usually elderly patients with osteoarthritis of the SIJs. With a hyperintense signal on STIR sequence, blood vessels crossing through the SIJs or surrounding ligament may seem and incorrectly be understood as active inflammation. Finally, the so called “coil effect” may result in false positive signals on STIR sequence. This finding is mainly seen outside of the periarticular region, closer to the coil-body interface, with similar effects on the adjacent soft tissue helping to distinguish this artifact from real alteration. The most important differential diagnoses in the spine are degenerative/mechanical lesions, blood vessels and hemangioma, fractures, and septic spondylitis/spondylodiscitis. In spondylosis, the bridging osteophyte or spondylophyte, with distinct direction and shape than those of the syndesmophyte, reveals the degeneration of the intervertebral disk (9). At first, they grow horizontally and then turn vertically, developing a “handle shape.” Corner-based lesions as seen by MRI, either BME or fat metaplasia, may also be visible with spondylophytes. Chronic back pain may be frequently seen in patients with degenerative disk disease. The resulting erosive osteochondrosis are the most typical degenerative lesions associated with BME. On MRI, typical findings are called Modic lesions, of which three types can be described based on their signal patterns (118). BME in these lesions is found in the area of the vertebral endplate eventually in conjunction with erosions, and accompanied by decreased height of the intervertebral disk. Similarly, endplate irregularities and erosive changes of the vertebral surface can be found in Scheuermann’s disease. Starting in childhood, it is frequently only recognized in the adulthood as a cause of chronic back pain. BME may be find around Schmorl’s nodules, which later may undergo fat metaplasia and sclerosis (119). As a classical differential diagnosis, DISH is characterized by wide, bulky osteophytes with concomitant ossification of the anterior longitudinal ligament. Distinction between AS and DISH is readily made by the radiographic appearance of the osteophytes, though they are characterized by similar pathological findings as new bone formation and BME may be present (120). Blood vessels typically present as a hyperintense signal on STIR sequence, in the center of the vertebral body, running from posterior to anterior. Likewise, hemangioma is an accumulation of vessels located within the vertebral body. These findings represent physiological abnormalities and not inflammatory lesions. Findings in infectious spondylitis/spondylodiscitis are similar to those in the SIJs. A special feature is the high frequency of abscesses located in the surrounding soft tissues. T1Gd sequence is usually needed to diagnose this complication (121). Finally, spinal fractures, sometimes resulting from minor trauma, may mimic axSpA exacerbation (122). Nondisplaced spinal fractures may not be easily recognized by CR in advanced AS.

## Summary

Despite major progress in the imaging diagnosis, CR continues to be the initial approach to evaluate patients with suspected axSpA. Early diagnosis and treatment are eased by the latest developments in imaging and classification of axSpA. MRI of the SIJs, capable to detect both active and structural lesions, has become vital in this role. Although BME, detected on the STIR or T2FS sequences, is considered essential for the definition of a positive MRI for axSpA, alone it lacks specificity and may be misleading. The contextual information provided by structural lesions, namely erosions and fat metaplasia, may enhance the confidence of the diagnosis. Novel MRI modalities further increase the sensitivity and specificity of conventional MRI, and deepen the understanding of the entire spectrum of the disease.

## Author Contributions

XB conceived the manuscript. NK collected and reviewed the literature, and wrote the main body of the manuscript. AR and XB critically reviewed the manuscript. All the authors approved the final version of the manuscript.

## Conflict of Interest Statement

The authors declare that the research was conducted in the absence of any commercial or financial relationships that could be construed as a potential conflict of interest.
